# A Stackable Triboelectric Nanogenerator for Wave-Driven Marine Buoys

**DOI:** 10.3390/nano12040594

**Published:** 2022-02-10

**Authors:** Hao Wang, Chuanqing Zhu, Weichen Wang, Ruijiang Xu, Pengfei Chen, Taili Du, Tingxi Xue, Zhaoyang Wang, Minyi Xu

**Affiliations:** 1 Dalian Key Lab of Marine Micro/Nano Energy and Self-Powered Systems, Marine Engineering College, Dalian Maritime University, Dalian 116026, China; hao8901@dlmu.edu.cn (H.W.); zcq@dlmu.edu.cn (C.Z.); wangweichen@dlmu.edu.cn (W.W.); xuruijiang@dlmu.edu.cn (R.X.); dutaili@dlmu.edu.cn (T.D.); tingxixue@gmail.com (T.X.); 15841832625@163.com (Z.W.); 2 Beijing Institute of Nanoenergy and Nanosystems, Chinese Academy of Sciences, Beijing 100085, China; chenpengfei@binn.cas.cn

**Keywords:** triboelectric nanogenerator, wave-driven, self-power, marine buoy

## Abstract

Marine distributed devices are essential infrastructure for exploring and utilizing the ocean. As the most common carrier of these devices, floating and submerged buoys are subject to a bottleneck of power supply. Recent progress in nanogenerators could convert the high-entropy marine kinetic energy (e.g., wave) robustly, which may form an in-situ power solution to marine distributed devices. This study is devoted to develop a stackable triboelectric nanogenerator (S-TENG), while each layer of it is made into multiple channels carrying PTFE balls in between Aluminum electrodes. In the experiments based on forced motion, the peak power density of the S-TENG reaches 49 W/m^3^, about 29% promotion from our previous benchmark. The S-TENG has also become less vulnerable to directional variation of the excitation, making its integration on various platforms more flexible in real conditions. In practice, the S-TENG has demonstrated its capability of powering LEDs as well as various sensors measuring salinity, temperature and acidity, which means the S-TENG could self-power many compact marine buoys.

## 1. Introduction

Marine distributed systems refer to a variety of floating and submerged devices deployed in the ocean and for the ocean. They carry out the critical tasks backing-up the marine economy (e.g., fishing, transportation, mining, etc.) and marine security (e.g., climate, defense, environment, etc.) [1,2]. For example, the in-situ marine data acquisition is usually achieved through surface (floating) or subsea (submerged) buoys. These buoys (like many other marine distributed systems) mainly rely on batteries or cables, which seizes their independence and flexibility, until recently being improved by the renewable energy technologies [3]. As of power unit volume, the water waves generally carry orders of magnitude of higher energy than that carried by wind or sunlight [4]. Therefore, it has been quite appealing if the marine distributed device could utilize the wave energy effectively [5].

On the other hand, wave energy should be categorized as a high-entropy renewable energy [6,7]. Unlike conventional energy, most renewable energy is not the simple stable flow of medium fluid (e.g., steam). Stable flow of medium fluid is the essential characteristic that drives almost all conventional power plants, (e.g., thermal/nuclear/hydroelectric plants). To work without the stable flow of medium fluid, the concurrent wind turbines provide a solution consisting of two critical elements: “control” and “large-scaling” [8,9]. Modern turbine control could greatly improve the stability challenged by the ever-changing wind velocity and wind direction [10]. Large scaling also contributes to the (output) stability of wind turbine and it is the underlying prerequisite for control to become economically feasible [11].

However, it is not exactly the same story for wave energy utilization [12,13]. As a matter of fact, wind flow is still a form of medium fluid flow (though not stable enough). In this sense, after being stabilized, wind energy becomes similar to conventional energy. Compared with wind, waves are accompanied by more randomness, as they are a vibration physically (instead of flow) [14]. Ocean engineering has adopted various wave spectrum (e.g., Bretschneider spectrum) to approximate complex waves [15]. Each random wave combines of dozens of regular waves (with a single frequency and the corresponding amplitude), while each of the waves are of the corresponding random phase. On observation of this, hydrodynamic control, though developing very fast [16,17], seems to be inadequate to address the wave by itself, especially when the carrier of wave energy converters are distributed marine structures that essentially need to be small scale.

Considering these, the unconventional, triboelectric nanogenerator (usually abbreviated as TENG) can become a promising candidate in wave energy utilization [18,19]. As for directly taking low frequency inputs, studies have revealed that there exists a critical frequency below which nanogenerators work superior to the electromagnetic generators [20]. Particularly, wave energy is highly concentrated within the frequency range of 0.05 Hz to 0.5 Hz [21], which is definitely a “sweet zone” for nanogenerators. This means, without adding expensive gear structures, the TENG can be a more promising pathway for wave energy conversion. In addition, triboelectric nanogenerators are naturally more robust to the wave environments, as a variety of them are made mostly by high polymer materials (not subject to marine corrosion).

Developing a high efficiency wave energy converter by implementing the TENG is very attractive, which has seen many progresses in the recent five years. These progresses consist of not only design innovation/material enhancement [22,23,24], but also power management/energy storage [25,26]. In a study by Zhonglin Wang’s team, under wave excitations, contact and separation of membranes within the TENG could power the wireless transmission of marine sensing data (at a distance of about 300 m) [27,28]. The authors’ previous work produced a buoy carrying sandwich-like TENG so that the peak power density reached 38 W/m^3^. It is worth noting that the TENG integrated buoy has gone through model tests in a professional wave basin (simulating the actual seastates) for the first time [29,30]. Besides these advances in demonstration application, what also grows is the understanding on the TENG. For instance, researchers found that a “hybrid” wave energy converter combining triboelectric generator with electromagnetic generator could greatly improve the output as well as the durability [31,32,33,34]. Recent progresses on the 3-D mathematical modeling of TENG’s electric field provide a better insight on the relationship between the TENG design and the corresponding performance [35,36]. On top of this work, this paper presents the authors’ recent endeavor for promoting the power density of wave-drive TENG.

## 2. Materials and Methods

**Figure 1:**
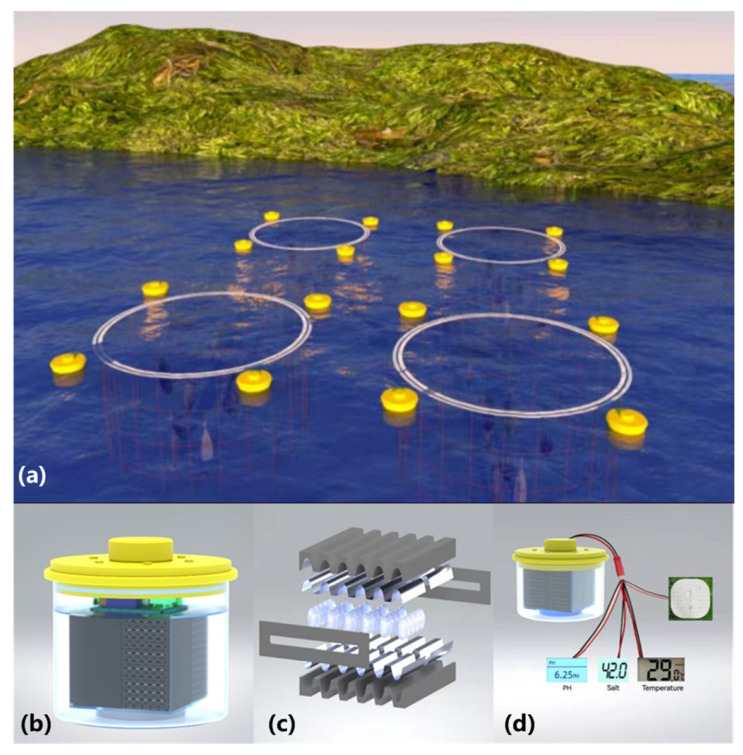
The TENG integrated buoy (**a**) A conceptual diagram of the surface buoys; (**b**) The make-up of the buoy; (**c**) The S-TENG integrated inside, with each layer in parallel connection; (**d**) Some demonstration applications for the S-TENG.

The initial intention of this study is to address the power supply for the in-situ marine data acquisition. Practically this is usually achieved through surface (floating) or subsea (submerged) buoys. To secure isotropy to incident wave and wind excitations, the geometry of the buoys are usually various modifications to cylinders. As shown in Figure 1a, the buoys are distributed over the surface of waters, measuring essential data for a marine “ranch” while marking the ranch (with LEDs) at night. As the buoys reach adequate quantity, a network of marine data acquisition can be formed, from which we can track series of meaningful clues within the waters.

Figure 1b presents the make-up of the buoy, which consists of an acrylic cylindrical frame and a nanogenerator inside (also shown in Supplemental Figure S1). The generator part is a compact module ready for integration onto other marine platforms where power supply is not easily accessible. In this study, a nanogenerator unit parallel connects 12 working layers to amplify the outputs, while the 12 layers are stacked in the same direction and sealed with an acrylic enclosure. The main body of the buoy (exposed to water) is sealed by screws and tape to be waterproof.

The TENG is produced with polylactic acid (PLA) material (through 3-D printing) so that multiple “channels” are created on one “layer” of the TENG. In fact, each layer is bounded by PLA plates (121 mm length, 118 mm width, 8.8 mm height) and PLA barrier sheets (118 mm length, 13.5 mm width, 2 mm thickness). These dimensions were determined from the comprehensive design practice. A pair of Aluminum electrodes of 55 mm width each are attached onto the PLA plate surfaces. The rotor in the device is many small balls (with a diameter slightly smaller than that of the channel) made with PTFE (poly tetra fluoroethylene) material. The 10.5 mm diameter PTFE balls are filled between the two plates and they could roll in the direction of inclination.

With the channels on each layer, the PTFE balls will not move in a chaotic way (which is found to be a negative factor for performance from our previous studies). As shown in Figure 1c, the channels on each layer bear a wave-like cross section, which is helpful in aspect of increasing effective contact in the balls’ rolling process. Through motions of the buoy, incident wave could excite the motion of these PTFE balls to a quite intensive level. The great difference in electronegativity leads to considerable electron loss for Aluminum (as it makes effective contact with PTFE). The wave-induced motion of the buoy makes the PTFE balls roll along the channels, where effective contacts take place. The S-TENG proves to be adequate for powering some (small-scale) marine devices, such as various sensors (i.e., temperature, salinity and acidity) and LEDs (see Figure 1d). Details of the demonstration application will be presented in Section 3.

A Keithley 6514 system electrometer measures the electrical outputs (i.e., short-circuit current, the transferred charge). The open-circuit voltage of the S-TENG unit cannot be measured directly, as its variation exceeds the allowable measuring range of the electrometer. The platform on which the forced motion tests are performed is a US-52 linear motor. The charging discharging tests for the sensors have been successfully conducted with the presence of a capacitor (220 μF, 16 V).

## 3. Results and Discussions

### 3.1. Output Performance of the S-TENG

**Figure 2:**
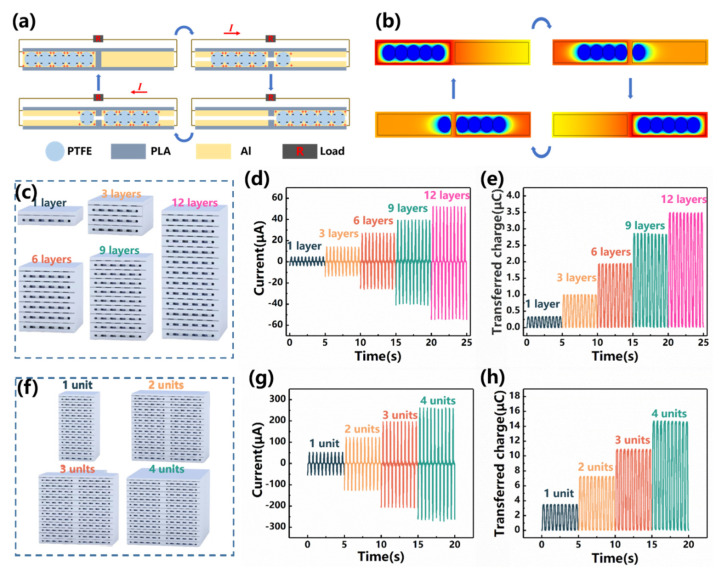
Working cycle and linearity examination for the TENG device (**a**) The schematic working cycle of the TENG; (**b**) The COMSOL visualized electric field; (**c**) Installing the S-TENG unit by stacking the multiple layers; (**d**) The short-circuit current variation with the layer number; (**e**) The transferred charge variation with the layer number; (**f**) Installing the nanogenerator module by combining 4 units; (**g**) The short-circuit current variation with the unit number; (**h**) The transferred charge variation with the unit number.

The working principle of the S-TENG is shown in Figure 2a. Four stages form a complete working cycle for the TENG. At the starting stage, the PTFE balls have accumulated adequate electrons on them (approximately a “steady state”) so that the electrons on the electrodes tend to concentrate on the opposite side. As it moves in one direction (e.g., to right in stage two), electrostatic induction will shift the electrons on the electrodes to the other direction (e.g., to left in stage two). The reciprocating motion of the PTFE balls along their channels is a repeated process of moving the electrons, creating an alternating electric potential across the electrodes. The electrodes are connected in a circuit containing necessary loads so that the motion of the PTFE balls induces currents in the outer circuit connecting the electrodes. The electric field consistently exerts additional resistance for the motion of the balls, which converts mechanical energy to electrical energy [37,38,39]. COMSOL multiphysics software visualizes the electric field variation, i.e., Figure 2b.

The performances of the S-TENG have been examined through systematic forced vibration tests. The S-TENG is forced to vibrate at a particular frequency and with a particular amplitude so that the corresponding electrical output can be measured. As the S-TENG involves parallel connection of layers and units, our experimental studies start from examining the linearity of performance (with respect to the number of connected layers/units).

Figure 2c depicts the gradual increase of TENG layers (from 1 to 12), while multiple layers are stacked (and parallel connected) and parallel connected in the circuit. At the frequency of 2 Hz and with the amplitude of 130 mm, the TENG yields a short circuit current of 4.68 μA. As the number of layers increases to 3, 6, 9 and 12 layers, the short circuit current increases correspondingly to 13.99 μA, 27.20 μA, 39.55 μA and 52.24 μA, which is a loosely linear increase (see Figure 2d). Similarly, at the frequency of 2 Hz and with the amplitude of 130 mm, the TENG yields a transferred charge of 0.33 μC. As the number of layers increases to 3, 6, 9 and 12 layers, the transferred charge increases correspondingly to 1.00 μC, 1.94 μC, 2.86 μC and 3.50 μC, which also yields a loosely linear correlation (see Figure 2e).

The nanogenerator module is installed by combining 4 units, as depicted in Figure 2f. At the frequency of 2 Hz and with the amplitude of 130 mm, as the number of units increases from 1 to 4, the short circuit current increases correspondingly to 52.24 μA, 121.43 μA, 196.91 μA and 261.792 μA (see Figure 2g), while the transferred charge increases correspondingly to 3.50 μC, 7.27 μC, 10.92 μC and 14.715 μC (see Figure 2h). Both the short circuit current and the transferred charge generally raise linearly with the unit number. The number of units is determined to be 4 to achieve a relative small-scale power module applicable for actual conditions.

**Figure 3:**
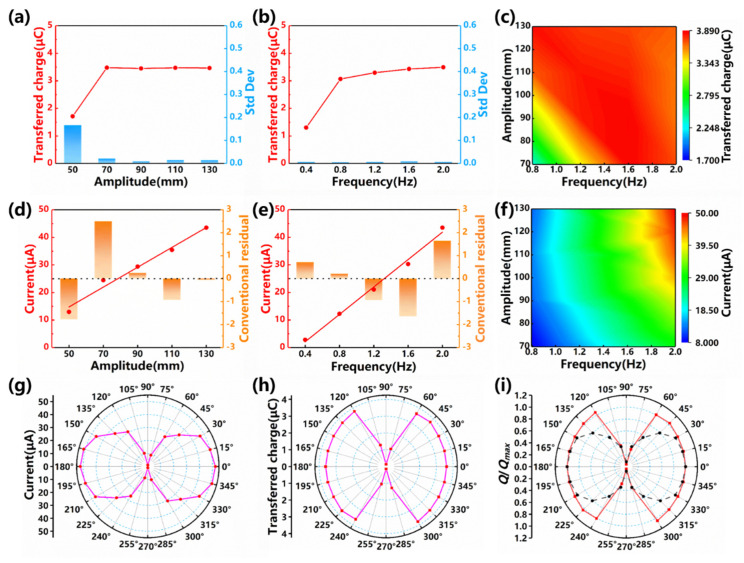
The performance of the S-TENG (1 units) under different vibration parameters. (**a**) The transferred charge variation with the vibration amplitude; (**b**) The transferred charge variation with the vibration frequency; (**c**) 3D contour of the transferred charge variation with the frequency and the amplitude; (**d**) The short-circuit current variation with the vibration amplitude; (**e**) The short-circuit current variation with the vibration frequency; (**f**) 3D contour of the short-circuit current variation with the frequency and the amplitude; (**g**) The azimuth dependence of the short-circuit current; (**h**) The azimuth dependence of the transferred charge; (**i**) The comparison between the S-TENG and the previous TENG on the azimuth map of the transferred charge (nondimensional).

As is shown in Figure 3a,b, the transferred charge yields an increase with the vibration amplitude and with the vibration frequency (time domain data is shown in Supplemental Figure S2). Obviously, there is a ceiling for the transferred charge (depending on the design specifications including material, size, geometry etc.), where the surface electrons in the S-TENG are fully activated and the PTFE balls are sufficiently charged. It can be noted that the only significant increase takes place as the vibration amplitude increases from 50 mm to 70 mm. The reason is that the length of one electrode (e.g., the left part) is 55 mm, the contact area could increase in the vicinity of this vibration amplitude (e.g., from 50 mm to 70 mm). Some dynamic details could give rise to the spike from 0.4 Hz to 0.8 Hz in Figure 3b. The PTFE balls are likely to “roll” instead of “slide” at 0.4 Hz, while “sliding” seems to be more relevant to the charge transfer. After 0.8 Hz, rolling has been largely eliminated so there is no more “spike”. Another possibility is that at 0.4 Hz, all the PTFE balls are more likely to finish their full “stroke”. Under higher frequency, they will not be able to finish their full “stroke”, which reduces the transfer charge at higher frequency.

Standard deviations for these measurements are found to be not significant. A 3-D contour of the transferred charge presents more clearly its variation with the frequency and the amplitude (see Figure 3c). The short-circuit current, on the other hand, correlates more closely with the vibration amplitude and the vibration frequency (see Figure 3d,e and Supplemental Figure S3). As the vibration amplitude raises from 50 mm to 70 mm, 90 mm, 110 mm, 130 mm, the short-circuit current increases from 12.98 μA, to 24.46 μA, 29.42 μA, 35.46 μA, 43.53 μA, yielding a quite linear trend (R2~0.995). Similarly, as the vibration frequency increases from 0.4 Hz to 0.8 Hz, 1.2 Hz, 1.6 Hz, 2.0 Hz, the short-circuit current raises from 2.84 μA, to 12.26 μA, 21.06 μA, 30.30 μA, 43.53 μA (R2~0.993). A 3-D contour of the short-circuit current presents more clearly its variation with frequency and amplitude (see Figure 3f).

As the studied TENG does not have isotropic channels (not symmetric with the *z*-axis), another important issue for it is the vibration direction. Experiments show that except for the “head sea” condition (in which the channel is in-line with the vibration direction, i.e., 0° degree or 180°), the output current will reduce, to different extent (as shown in Figure 3g). While the transferred charge is also subject to the directional reduction, the reduction is only significant within a third of the azimuth (i.e., 60°~120°, 240°~300°, see Figure 3h). This is improved from the previous study, as compared in Figure 3i, an azimuth map of the (nondimensional) transferred charge. Given these data, the directional reduction looks quite undesirable. However, floating structures are not only excited by wave, but also by wind and other forces. For example, the inevitable difference between wave azimuth and wind azimuth will induce non-neglectable roll and sway motion (perpendicular to the wave direction). Therefore, even as wave comes in the undesirable direction (e.g., 90°), the S-TENG could utilize the roll and sway motion (they are in 0°/180° azimuth, as discussed in Section 3.2).

**Figure 4:**
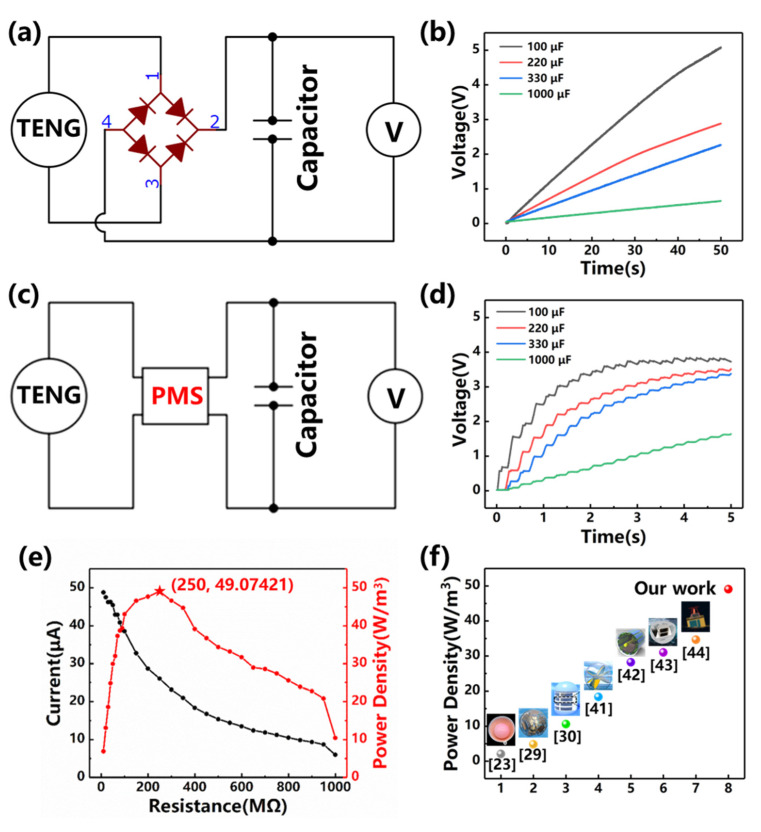
Charging the capacitance with the TENG (**a**) The diagram depicting the charging circuit; (**b**) The time series (within 50 s) of the voltages during charging with various capacitances; (**c**) The diagram depicting the charging circuit with the PMS; (**d**) The time series (within 5 s) of the voltages during charging with various capacitances (with the PMS); (**e**) The output current and the power density of the TENG with respect to resistance in the circuit; (**f**) A comparison of the output power density with other wave-driven TENGs [23,29,30,41,42,43,44].

Figure 4a presents the original charging circuit without any power management system (PMS), on which a series of 50 s charging experiments are performed (see Figure 4b). After 50 s charging, the capacitor reaches 5.09 V, 2.89 V, 2.27 V, 0.65 V with the capacitance of 100 μF, 220 μF, 330 μF, 1000 μF, respectively. In practice, the charging process will be boosted with appropriate power management. The power management circuit is similar to that in the reference [40]. Figure 4c presents the modified charging circuit (implementing a Power Management System) on which a series of 5 s charging experiments are performed (see Figure 4d). After 5 s charging, the capacitor reaches 4.12 V, 3.52 V, 3.37 V, 1.64 V with the capacitance of 100 μF, 220 μF, 330 μF, 1000 μF, respectively. It needs to be noted that the capacitance of 1000 uF will reach saturation in about 15 s when the voltage reaches 2.3 V.

The output current, as well as the power density of the S-TENG under various load resistance is plotted in Figure 4e. The peak power density *D_max_* and the average power density *D_ave_* can be evaluated by the following equation:

(1) $$\{\begin{array}{c} D_{max}=\frac{I^{2}R}{V}\\ D_{ave}=\frac{\int_{0}^{T}I^{2}Rdt}{T\cdot V} \end{array}$$

*I* is the current, *R* is the load resistance. *T* is the cycle period and *V* is the device volume. The peak power reaches 49.07 W/m^3^ when the load resistance equals the internal resistance (around 250 MΩ). The reduction of power at non-optimal load resistance is more flat than that in our previous study, indicating that the TENG could accommodate more sorts of loads. In general, compared with other wave-driven TENGs, this study has achieved a new benchmark in power density [23,29,30,41,42,43,44].

### 3.2. Demonstration Application of the S-TENG

The ocean environment is a mixture of complex dynamics. What dominates the performance of a floating structure is wave, wind and current. Usually, wave loads are comparable with wind loads, while they are an order of magnitude larger than current loads. Therefore, the interaction between the floating structure and waves (commonly referred to as hydrodynamics), should be understood.

**Figure 5:**
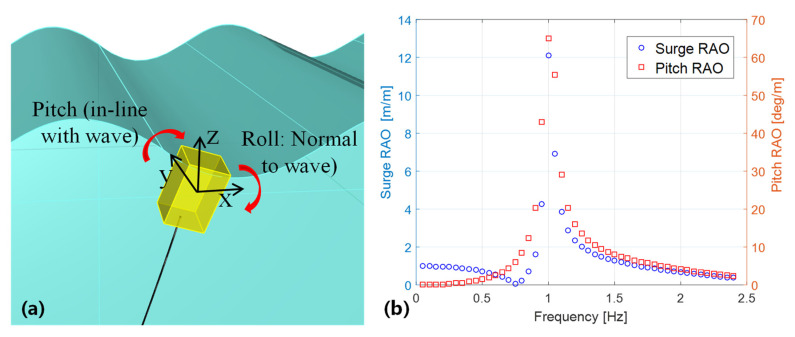
Motion analysis for the buoy (**a**) The effective rotation of the buoy; (**b**) The response amplitude operator of the buoy.

In the case of the S-TENG, its output performance depends on the pitch/roll and surge/sway motion (see Figure 5a, contribution from roll and sway have been explained in Section 3.1). The pitch and surge motion are mainly contributed by wave direct excitation (namely Froude-Krylov force and diffraction force). Yet for roll and sway, x-z symmetry structures (e.g., the buoy) are not subject to direct wave excitation. Their roll and sway are mainly attributed to the parametric excitations due to the nonlinear effects of the variation of the structure’s submerged volume. The parametric excitation also adds to the pitch and surge motion, though it is usually not as large as the direct excitation. To address the basics for designing the buoy, the authors have simplified the hydrodynamics. In that case, the wave-induced motion of a floating structure is described by the following equation [45,46,47]:

(2) $$\left[M+A\left(\infty \right)\right]\ddot{\xi }\left(t\right)+B_{l}\dot{\xi }\left(t\right)+B_{q}\dot{\xi }\left(t\right)\left|\dot{\xi }\left(t\right)\right|+\int_{0}^{t}h\left(t-\tau \right)\ddot{\xi }\left(t\right)d\tau +K\xi \left(t\right)=f_{w}\left(t\right)$$

where [*ξ*] is the multi-degree of freedom motion vector, [*M*] denotes the 6 × 6 mass matrix, [*A*(∞)] represents the 6 × 6 added mass matrix at infinite frequency, *B_l_* is the 6 × 6 linear viscous damping matrix, *B_q_* is the 6 × 6 quadratic viscous damping matrix, [*K*] denotes the 6 × 6 stiffness matrix (the mooring forces are incorporated into the *Kξ*(*t*) term) and [*f_w_*] is the wave excitations.

The dimension of the buoy is 14.6 cm (length) × 13.6 cm (width) × 24.5 cm (height). The length and width is determined so that S-TENG units can be fitted (also considering the buoy outer structure has 3 mm thickness). The height is determined so that: (1). The buoy should have a natural frequency of around 1 Hz (see Figure 5b). The natural frequency of 1 Hz is settled from our previous studies. This requirement determines the draft of the buoy; (2). The buoy should have adequate free board (height above the water line) to secure its stability. In addition, the buoy should have adequate space for the S-TENG units and the ballast. This requirement determines the free board of the buoy. The height is the sum of the draft and the free board.

The S-TENG (about 2.3 kg) is installed into the buoy structure (about 0.8 kg) ballasted with an iron plate (about 1.1 kg) at the bottom of the buoy, reaching a total mass of 4.2 kg. Moored in the test basin (details refer to Supplemental Figure S4), the whole system yields a static draft of about 23 cm. Based on the multi-degree of freedom equation of motions (1), the response amplitude operator (RAO) of the buoy can be analyzed with the common frequency domain, hydrodynamic software WAMIT (see Figure 5b).

**Figure 6:**
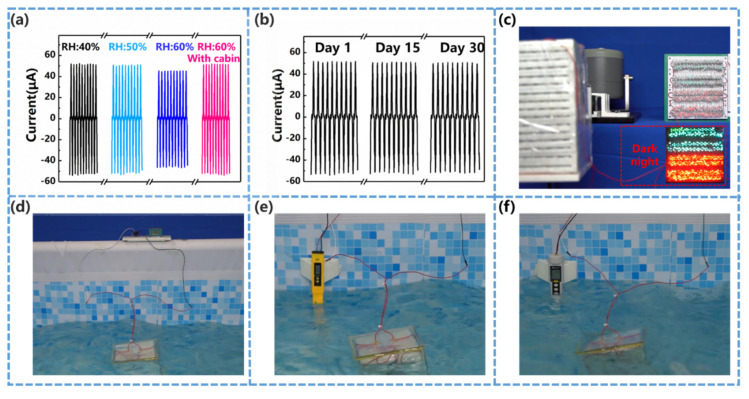
Demonstration applications (**a**) Sensitivity of the S-TENG, with respect to the relative humidity; (**b**) Durability of the S-TENG; (**c**) The S-TENG lighting 350 LEDs; (**d**) The S-TENG driving temperature sensor; (**e**) The S-TENG driving PH sensor; (**f**) The S-TENG driving salinity sensor.

A series of examinations have primarily verified the robustness of the S-TENG in marine environment. For example, as the relative humidity is raised from 40% to 60%, the output performance (i.e., current) yields a reduction of around 13% (see Figure 6a), which is acceptable for marine equipment. In fact, it is believed that the S-TENG implemented in a waterproof module is subject to even less influence from the environmental humidity. Repeated experiments throughout a month reveal that the S-TENG’s performance is quite stable (see Figure 6b).

On top of these, some common functions of (small-scale) marine devices are tested on the S-TENG. In the demonstration presented by Figure 6c and Video S1, the S-TENG converts the vibrations (simulated by a linear motor) to alternating currents that easily light up 350 LEDs (the function of navigation buoy essentially). Figure 6d through Figure 6e demonstrate the wave-drive S-TENG as an effective power source for various marine sensors. With the power from a single unit S-TENG (to fit the dimension of typical CTD profilers), a temperature sensor could make one measurement every 10 s (see Figure 6d and Video S2), a PH sensor could make one measurement every 2 min (see Figure 6e and Video S3) and a salinity sensor could make one measurement every 5 min (see Figure 6f and Video S4), respectively. The measuring step can be improved further, though it is not the bottleneck (practically the measuring durability is usually more critical for marine sensors). In fact, most marine sensors require a similar level of power supply, indicating that the S-TENG has passed the threshold of backing-up a variety of marine sensing (measurement) tasks (e.g., radiation, oxygen, pressure and conductivity).

## 4. Conclusions

This study concerns itself with developing a stackable triboelectric nanogenerator (namely, the S-TENG) for wave-driven marine buoys. On top of the previous studies, the authors have modified the structure of the multi-channel, multi-layer S-TENG so that its internal space is well utilized. Forced motion tests in the laboratory have observed a loosely linear increase of the output performance with the number of layers as well as with the number of units. The S-TENG records a peak power density of 49 W/m^3^, about 29% promotion from our previous work, making it a new benchmark in aspect of power density compared with other wave-driven TENGs. The S-TENG has become less vulnerable to directional variation of the excitation, making it more robust for integration on boarding various marine platforms. The S-TENG has demonstrated its capability of serving the navigation buoy as well as powering marine sensor measuring salinity, temperature and acidity. Considering the power requirements of small-scale marine devices, the wave-driven S-TENG could form an effective in-situ power module to achieve self-powered marine buoys. The methodology of the study considers hydrodynamics (from wave to buoy motion) and electrodynamics (from buoy motion to electrical outputs) separately. This means that it has not reached the level of wave-to-wire modelling, which is the main shortage we will overcome in further work.

## Data Availability

The data presented in this study are available on request from the corresponding author.

## Supplementary Materials

The following are available online at https://www.mdpi.com/article/10.3390/nano12040594/s1, Figure S1: Photograph of the S-TENG, Figure S2: The transferred charge time series, Figure S3: The short circuit current time series Figure S4: Photograph of the S-TENG in the test basin, Video S1: 350 LEDs lighted up by the S-TENG, Video S2: The S-TENG driving temperature sensor in water, Video S3: The S-TENG driving PH sensor in water, Video S4: The S-TENG driving salinity sensor in water.
