# Supplementary material for: A Stackable Triboelectric Nanogenerator for Wave-Driven Marine Buoys
Source: Nanomaterials (Basel). 2022 Feb 10;12(4):594. doi: 10.3390/nano12040594 (PMC8877952; doi:10.3390/nano12040594)
Supplement: Supplementary file 1 [file nanomaterials-12-00594-s001.zip › nanomaterials-1568456-supplementary figures.pdf]

nanomaterials

Supplementary Materials

A Stackable Triboelectric Nanogenerator for Wave-Driven Marine Buoys

Hao Wang [1,†], Chuanqing Zhu [1,†], Weichen Wang [1,†], Ruijiang Xu [1], Pengfei Chen [2], Taili Du [1], Tingxi Xue [1], Zhaoyang Wang [1] and Minyi Xu [1,*]

1 Dalian Key Lab of Marine Micro/Nano Energy and Self-powered Systems, Marine Engineering College, Dalian Maritime University, Dalian, 116026, China; hao8901@ dlmu.edu.cn (H.W.); zcq@dlmu.edu.cn (C.Z.); wangweichen@dlmu.edu.cn (W.W.); xuruijiang@dlmu.edu.cn (R.X.); dutaili@dlmu.edu.cn (T.D.); tingxixue@gmail.com (T.X.); 15841832625@163.com (Z.W.)
2 Beijing Institute of Nanoenergy and Nanosystems, Chinese Academy of Sciences, Beijing 100085, China; chenpengfei@binn.cas.cn (P.C.)
* Correspondence: xuminyi@dlmu.edu.cn; Tel.: +86-18941134769
† These authors contributed equally to this work.

Citation: Wang, H.; Zhu, C.; Wang, W.; Xu, R.; Chen, P.; Du, T.; Xue, T.; Wang, Z.; Xu, M. A Stackable Triboelectric Nanogenerator for Wave-Driven Marine Buoys. *Nanomaterials* **2022**, *12*, 594. https://doi.org/10.3390/nano12040594

Academic Editor: Fabrizio Pirri

Received: 7 January 2022
Accepted: 7 February 2022
Published: 10 February 2022

Publisher's Note: MDPI stays neutral with regard to jurisdictional claims in published maps and institutional affiliations.

Copyright: © 2022 by the authors. Licensee MDPI, Basel, Switzerland. This article is an open access article distributed under the terms and conditions of the Creative Commons Attribution (CC BY) license (https://creativecommons.org/licenses/by/4.0/).

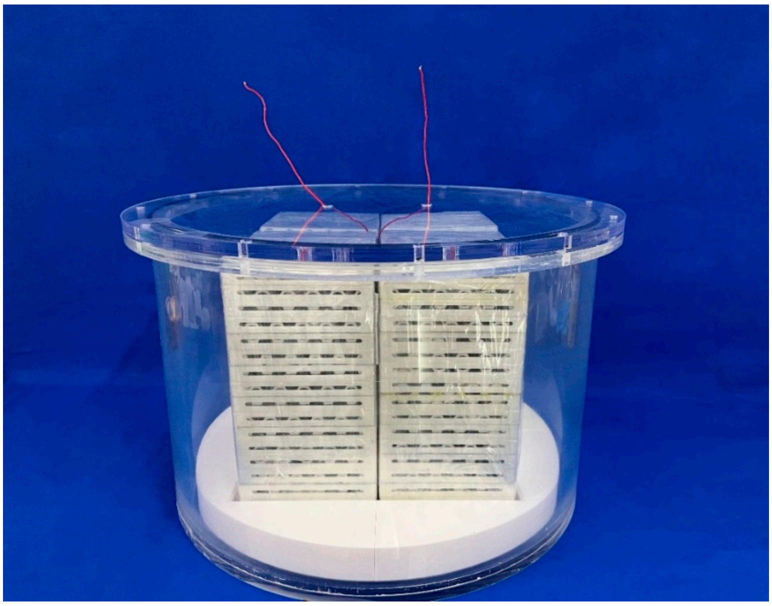

Figure S1. Photograph of the S-TENG.

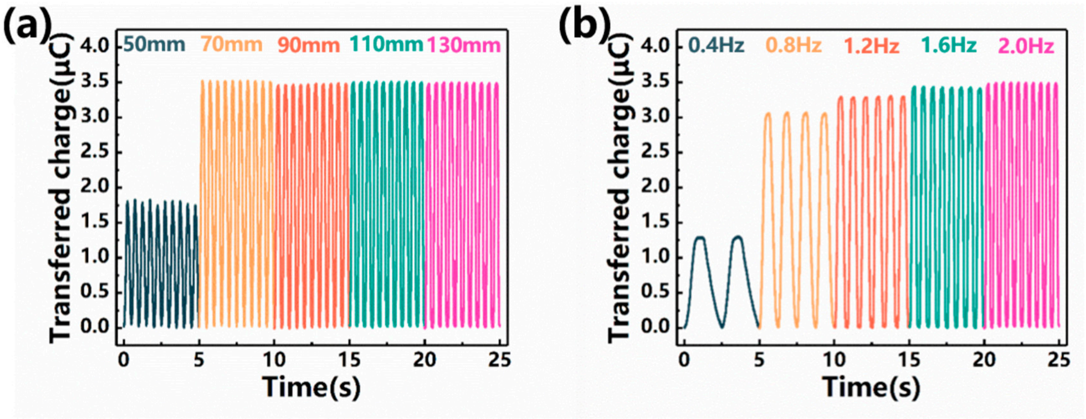

Figure S2. The transferred charge time series (**a**) variation with the vibration amplitude (**b**) variation with the vibration frequency (corresponding to Figure 3 (a,b)).

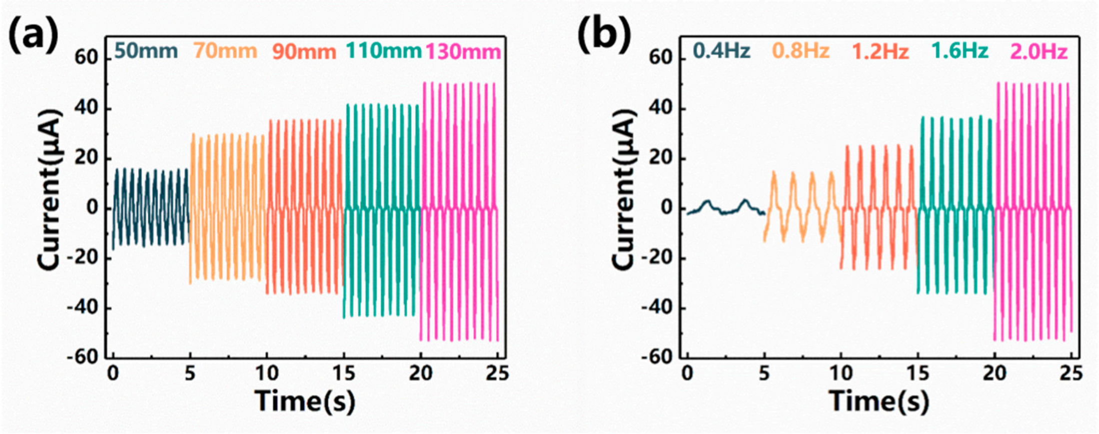

Figure S3. The short circuit current time series (**a**) variation with the vibration amplitude (**b**) variation with the vibration frequency (corresponding to Figure 3 (d, e)).

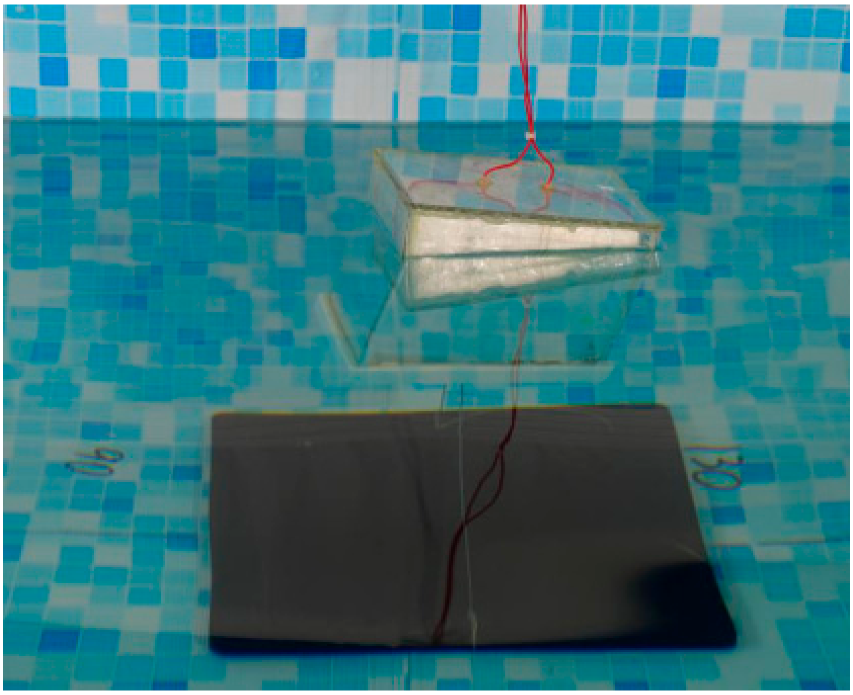

Figure S4. Photograph of the S-TENG in the test basin.

The following demo videos are also uploaded:
Supplementary Video S1: 350 LEDs lighted up by the S-TENG.
Supplementary Video S2: The S-TENG driving temperature sensor in water
Supplementary Video S3: The S-TENG driving PH sensor in water
Supplementary Video S4: The S-TENG driving salinity sensor in water
